# Experiences in the care of Indigenous people in hospital settings: challenges for clinical management

**DOI:** 10.1590/0034-7167-2024-0467

**Published:** 2026-03-16

**Authors:** Alidio Vieira Nunes Duarte, Vanessa Alves Mendes, Carolina Giordani da Silva, Mara Regina Rosa Ribeiro, Gímerson Erick Ferreira

**Affiliations:** I Hospital Universitário Júlio Müller. Cuiabá, Mato Grosso, Brazil; II Universidade Federal de Mato Grosso. Cuiabá, Mato Grosso, Brazil

**Keywords:** Clinical Management, Health of Indigenous Populations, Integrality in Health, Indigenous Culture, Indigenous People., Gestión Clínica, Salud de las Poblaciones Indígenas, Integralidad en Salud, Cultura Indígena, Pueblos Indígenas.

## Abstract

**Objectives:**

to analyze the experiences of hospital care professionals in providing care to Indigenous individuals, from the perspective of clinical management.

**Methods:**

a qualitative, descriptive-exploratory study using interviews guided by the Critical Incident Technique, conducted with 18 professionals from a federal university hospital in the Central-West region of Brazil. Data were analyzed using IRaMuTeQ^®^ software and thematic content analysis.

**Results:**

five semantic classes emerged, organized into two categories, highlighting challenges such as cultural and linguistic barriers and the lack of intercultural training. These factors compromise continuity of care and communication with patients, weakening the implementation of clinical management.

**Final Considerations:**

clinical management, when aligned with cultural specificities, proves to be a strategic approach to improving hospital care for Indigenous populations. The study contributes to the field of Nursing by emphasizing the importance of intercultural training and care coordination, promoting integrated, equitable, responsive, and culturally sensitive approaches that address the real needs of these communities.

## RESUMEN

**Objetivos:**

analizar la experiencia de los profesionales de la salud en la atención hospitalaria al cuidado de personas indígenas, desde la perspectiva de la gestión clínica.

**Métodos:**

estudio cualitativo, descriptivo-exploratorio, basado en entrevistas guiadas por la Técnica del Incidente Crítico, con 18 profesionales de un hospital universitario federal en la región Centro-Oeste de Brasil. Los datos fueron analizados con el apoyo del *software* IRaMuTeQ^®^ y mediante análisis de contenido temático.

**Resultados:**

emergieron cinco clases semánticas organizadas en dos categorías, que evidencian desafíos como barreras culturales y lingüísticas, además de la falta de formación intercultural, lo que compromete la continuidad del cuidado y la comunicación con el paciente, debilitando la implementación de la gestión clínica.

**Consideraciones Finales:**

la gestión clínica, aliada a las especificidades culturales, se muestra como una estrategia clave para mejorar la atención hospitalaria a la población indígena. El estudio aporta a la Enfermería al destacar la importancia de la formación intercultural y la coordinación del cuidado, favoreciendo enfoques integrados, equitativos, sensibles y culturalmente adecuados a las necesidades reales de estos pueblos.

## INTRODUCTION

The Indigenous population in Brazil represents approximately 0.83% of the total population, with around 1,693,535 individuals^(1)^. This population faces various forms of social inequality and achieving universal and equitable access to healthcare services remains a significant challenge^(2)^.

Despite progress in recent years with the implementation of the “*Política Nacional de Atenção à Saúde dos Povos Indígenas”* (*“PNASPI”*, National Policy for Indigenous Peoples’ Health Care), obstacles persist in discussions surrounding the real health needs of this population. Issues such as inadequate reception and lack of continuity in care within the healthcare network are still evident^(3)^. This scenario highlights the urgent need for a deeper debate on the management of Indigenous health within Brazil’s “*Sistema Único de Saúde*” (*“SUS”*, Unified Health System), particularly regarding access to medium and high-complexity services.

In terms of hospital care, it is imperative to adapt services to the realities of Indigenous populations, recognizing legitimate and unique aspects of their needs and health determinants, with integrated systems and coordination with the territories where they live. In this context, proactive movements in Indigenous health care have emerged, aligning with principles of clinical management^(4)^, such as specialized matrix support and individualized therapeutic projects, which are strategic technologies for reference teams. These approaches resonate with Madeleine Leininger’s Theory of Transcultural Care, which advocates for care actions grounded in the cultural frameworks of the person being cared for, promoting practices that are congruent with the diverse health demands^(5)^.

Together, these approaches support an expanded conception of Indigenous hospital care, one that goes beyond the biological dimension and incorporates subjective, cultural, and social elements. This promotes continuity and comprehensive care across the various points of the *“Rede de Atenção à Saúde”* (*“RAS”*, Health Care Network)^(6)^. Such a perspective demands professionals who are trained to work in culturally diverse contexts, which is essential to ensuring the quality of care^(7)^.

In this regard, clinical management, an approach to care conceptualized in Brazil by Eugênio Vilaça Mendes, offers a unique opportunity to address the gaps that permeate hospital care for Indigenous individuals. This is because the model proposes a system of micro-management technologies applicable to *“SUS”* ^(6)^. It is grounded in the integration of management, care, and education to consolidate its practices, and is guided by principles that include: orientation toward health needs and comprehensive care; assurance of quality and safety in service delivery; valuing diverse knowledge and practices to solve health problems, sharing of power and co-responsibility among those involved in health production, continuous education for individuals and organizations, focus on outcomes that improve health and quality of life, and a commitment to transparency and collective accountability^(8)^.

In the more complex cases of Indigenous health care, services are often referred to other points within the *“RAS”*, located in urban centers, which serve as complementary to the care provided in the villages^(9)^. However, various conditions of vulnerability experienced by Indigenous peoples limit the effectiveness of health care, whether due to organizational, geographic, and/or cultural factors, such as transportation difficulties, language barriers, lack of adequate infrastructure, and shortage of trained professionals, among others^(10)^.

Therefore, for health services to integrate traditional practices and Indigenous knowledge, and for the various services within the *“RAS”* to understand and incorporate the real needs of this population into their practices, it is essential to foster articulation between Indigenous and biomedical knowledge^(11)^. This integration is crucial to ensuring comprehensive, equitable, and effective care. Given this context, the central question of this study emerges: How can the experiences of hospital care professionals in providing care to Indigenous individuals be analyzed through the lens of clinical management?

## OBJECTIVES

To analyze the experiences of hospital care professionals in providing care to Indigenous individuals from the perspective of clinical management.

## METHODS

### Ethical Aspects

This study is part of the matrix project entitled “Artifacts for the implementation of clinical management in a university hospital”, approved by the local Research Ethics Committee (CEP), under opinion number 6.080.13 and registered with *“Plataforma Brasil”* under CAAE number 09495919900005541. The study complies with the guidelines of Resolution No. 466, dated December 12, 2012, of the *“Conselho Nacional de Saúde”* (National Health Council). Participation was voluntary and took place upon signing the Free and Informed Consent Form (FICF). To ensure confidentiality and anonymity, participants’ statements were coded using the letter E followed by a cardinal number.

### Type of study

This is a descriptive-exploratory study with a qualitative approach, aiming to understand the human phenomenon through meanings, motives, aspirations, beliefs, values, and attitudes^(12)^. The article follows the guidelines of the *Consolidated Criteria for Reporting Qualitative Research* (COREQ).

### Study Setting

The study was conducted in a Federal University Hospital (HUF), managed by the *“Empresa Brasileira de Serviços Hospitalares”* (*“EBSERH”*, Brazilian Hospital Services Company), located in the Central-West region of Brazil. The units selected for the research were the maternal and child inpatient clinic and the outpatient departments (pediatrics, infectious diseases, wound care, and stomatherapy), due to their higher demand for services provided to Indigenous individuals.

### Data sources

A total of 18 health professionals from the multidisciplinary team participated in the study, including nurses, physicians, nursing technicians, a psychologist, a nutritionist, and a social worker, all with experience in providing care to Indigenous individuals. The sample was selected using the snowball sampling technique, in which key participants refer others^(13)^.

Inclusion criteria were professionals from the multidisciplinary team with an active employment status and experience in caring for Indigenous individuals in the clinics and outpatient departments of the hospital in question. Professionals who were on leave or vacation during the data collection period were excluded.

### Data collection and organization

Data were collected between July and August 2023 through semi-structured interviews guided by the Critical Incident Technique (CIT). This technique is widely used in health-related studies as it enables participants to recall memories of significant events and observations, facilitating the understanding of behaviors and lived experiences^(14)^.

**Figure 1:**
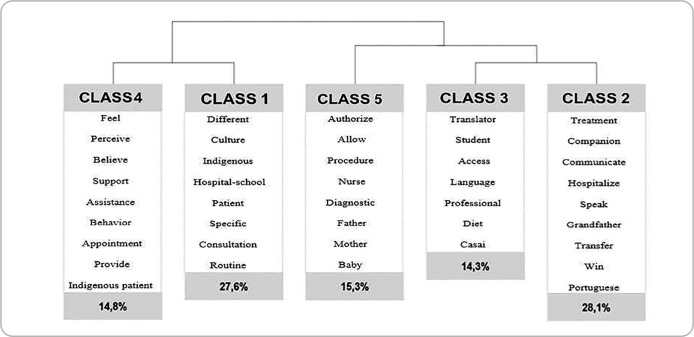
Semantic classes related to experiences in the care of Indigenous people, 2023

The interviews were audio-recorded and conducted by one of the researchers, a specialist in Indigenous health, with an average duration of 15 minutes. The empirical material was fully transcribed using the online transcription tool in Microsoft Word^®^ and subsequently processed using IRaMuTeQ^®^ software (*Interface de R pour les Analyses Multidimensionnelles de Textes et de Questionnaires*), which supported the qualitative analysis. To construct the classes, the Reinert Method was applied, utilizing 80% of the textual corpus, resulting in the grouping of 196 textual segments extracted from the transcripts. The Descending Hierarchical Classification (DHC) method was then used to generate semantically related classes, hierarchically represented in a dendrogram (Figure 1). This structure guided the systematization of the content, with the analytical focus placed on interpreting the meanings present in the participants’ narratives, which informed the development of the central thematic categories.

### Data analysis

Data analysis was conducted using Thematic Content Analysis^(15)^, following three stages: (I) data organization; (II) material exploration, with information grouped into categories; and (III) critical reflection for interpreting the data in light of the theoretical framework. This approach enabled the identification of health professionals’ experiences in providing care to Indigenous individuals within the hospital setting, from the perspective of clinical management.

## RESULTS

Among the study participants, 78% (14) were female and 22% (4) male, with an age range between 31 and 40 years (44.44%). Regarding professional background, 22.22% (4) were physicians, 33.33% (6) nurses, 27.78% (5) nursing technicians, 5.56% (1) psychologist, 5.56% (1) social worker, and 5.56% (1) nutritionist. The predominant educational level was higher education with a Lato Sensu specialization (50%). The length of professional experience varied, with 38.89% having between 11 and 15 years of experience. Participants mainly worked in outpatient units (38.89%) and in the pediatric clinic (22.22%).

The qualitative analysis of the data, supported by the IRaMuTeQ software, generated five classes organized into two axes. The professionals’ narratives enabled the construction of two central thematic categories related to the challenges present in their experiences of caring for Indigenous individuals: “Coordination and continuity of care” and “Cultural integration and adaptation of care processes”. Figure 1 illustrates the relationships between these classes and the identified thematic axes. It is worth noting that, although the software processing assisted in organizing the data, the analysis focused on the meanings expressed by the participants, without being limited to the frequency or distribution of textual segments.

In this context, two categories emerged from this analysis, the first titled “Challenges in the coordination and continuity of care for Indigenous people”. In this category, the professionals’ narratives reveal the main obstacles faced in ensuring continuity of care for the Indigenous population, particularly regarding intercultural communication and the integration between different levels of care within the *“RAS”*. The speeches point to the presence of linguistic and cultural barriers, compounded by weak information exchange and the absence of coordinated care pathways, especially during transitions between primary and tertiary care services. Notably, several narratives highlight the Indigenous man as the primary communication link, a hierarchical pattern common in certain ethnic groups due to his command of other languages. This dynamic further limits direct communication with the patient, compromising the understanding of their actual needs and, consequently, the quality of care and equity in service delivery. Chart 1 presents representative text segments that illustrate the emerging subcategories.

**Chart 1:** Representative text segments of Category 1, “Challenges in the coordination and continuity of care for Indigenous people”, organized by semantic class

CATEGORY 1 - “Challenges in the coordination and continuity of care for Indigenous people”	
Subcategories I (Classes 2 e 3): Need to adapt communication practices. Difficulties in care transition and continuity of assistance across different points in the healthcare network.	*The patient and their companion were both Indigenous, and they only communicated with each other. They didn’t understand what we were saying, and we didn’t understand what they were saying either. So, that created a challenge in our work.* (E04)
	[...] *when there were communication difficulties, I think that really affected the quality of care.* (E03)
	[...] *I think it would make our work and their lives much easier if they had more organized local support, you know? If there were a way to communicate with the doctor or nurse from their area, if we could have that connection between professionals from the tertiary care network and the primary care network* [...]. *It would really improve care for these patients, because I could pass on recommendations to the local professional, and they could provide direct assistance to the patient in their region.* (E11)
	*The difficulty we have with Indigenous patients is that when they’re admitted to the hospital, they usually don’t understand Portuguese and struggle to understand the care being provided.* (E13)
Subcategory II (Class 5): Importance of procedural and administrative aspects in the implementation and coordination of care.	*The mother didn’t speak to us, and the father was very difficult, he didn’t accept the treatment, and it took a lot of effort, talking to him* […]. (E10)
	*The father came along, but when we needed to urgently perform a procedure on the child, the nurse couldn’t proceed because the mother wouldn’t allow it, only with the father’s authorization.* (E17)
	*The mother wouldn’t allow it because the father wasn’t present. She couldn’t authorize it without the father being there.* (E17)

The second category, titled “Challenges in cultural integration and adaptation of care processes for Indigenous people”, encompasses the meanings attributed by professionals to the difficulties in incorporating Indigenous cultural elements into hospital care. The narratives highlight a lack of specific training for providing care to this population, as well as feelings of insecurity, dissatisfaction, and even helplessness in the face of institutional unpreparedness to address cultural differences. In many cases, the care provided follows standardized models that overlook the values, beliefs, and traditional practices of Indigenous patients. The excerpts presented in Chart 2 reveal the complexity of these experiences and how they affect both the quality of care and the relationship between healthcare professionals and patients.

**Chart 2:** Representative text segments from Category 2: “Challenges in cultural integration and adaptation of care processes for Indigenous people”, organized by semantic class.

CATEGORY 2 - “Challenges in cultural integration and adaptation of care processes for Indigenous people”	
Subcategory I (Class 1): Importance of recognizing and respecting Indigenous culture in the hospital setting.	*They have a culture different from ours, and sometimes we struggle to adapt the hospital routine to their culture.* (E05)
	*We care for them as if they were any other patient, following our usual routine, but we lack the training and specific care practices for Indigenous patients* [...] *it’s very different from our culture.* (E05)
	*You can tell they don’t show an understanding of what’s being proposed, and the team - speaking of the professional team - also lacks the sensitivity to grasp it.* (E09)
Subcategory II (Class 4): Challenges faced by professionals in providing adequate care.	*It really stuck with me because we didn’t get much support, and I’m not even sure if it was because the institution didn’t want to provide it.* (E05)
	*I noticed people’s behavior, it’s not exactly negligence, but rather a superficial kind of care, less communicative, rough around the edges, drier, with little conversation and minimal interaction with the patient.* (E09)
	*We have a lot of difficulty understanding each other during consultations. I’m not always confident that what I prescribe is truly understood, I still feel unsure whether they’re really grasping what I’m trying to convey.* (E11)
	*We don’t understand their culture very well, sometimes not even their language, and we end up feeling powerless.* (E10)

## DISCUSSION

Indigenous health care in hospital settings is marked by complex challenges faced by health professionals, reflecting the need for a more integrated approach adapted to the cultural specificities of Indigenous populations. These challenges go beyond basic access to health services, encompassing key aspects of intercultural communication and care coordination-both essential to ensuring comprehensive and high-quality care^(16)^. In this context, clinical management requires the involvement of well-articulated interprofessional teams capable of integrating diverse forms of knowledge and sharing responsibilities in the care of Indigenous populations^(6)^.

The research findings highlight that effective communication and coordination across different levels of care are critical factors for the effectiveness of hospital-based Indigenous health services. The analysis revealed difficulties in care transitions and continuity of assistance, as reflected in participants’ accounts of the complexity involved in communication among various health services. From the perspective of clinical management, these elements are necessary to ensure quality care and continuity, demanding an integrated interprofessional approach that promotes information exchange and the adaptation of practices to the cultural specificities of patients. This process involves navigation support within the health system, encompassing both clinical coordination, such as referrals and information transfers, and logistical coordination, including support, transportation, and financial matters^(6)^.

In this sense, it becomes essential to implement strategies to overcome linguistic and cultural barriers, such as the use of translators and the adaptation of communication materials, taking into account the cultural and linguistic particularities of each Indigenous ethnic group. The development of bilingual educational materials, with the participation of Indigenous professionals and community leaders, may represent a concrete and feasible measure within the hospital context. Such initiatives contribute to improving communication across different levels of care, facilitating the appropriate transfer of information and responsibilities, and ensuring that the follow-up of Indigenous patients occurs in a coordinated and culturally sensitive manner.

However, when addressing hospital-level care for Indigenous populations, the findings of this study point to gaps in care coordination, particularly in the transition between different levels of care and in the counter-referral process to villages or territories. This lack of coordination mechanisms may negatively impact continuity of care, hindering integration between hospital services and the local health network. This scenario underscores the need for proactive network-based strategies focused on implementing transitional care that is sensitive and aligned with the cultural specificities of each Indigenous ethnic group^(5,17)^ and reaffirms the importance of interprofessional collaboration as a central element in clinical management.

To address the identified gaps, clinical management emerges as a potentially effective model, as its guiding principles are grounded in protocols and guidelines that facilitate communication and integration across services at various points of care within the *“RAS”* ^(4)^. In theory, this model promotes the formation of dedicated teams and the inclusion of Indigenous communities in the development and monitoring of strategies, aligning with the need for specific clinical guidelines to improve coordination and continuity of care.

From the perspective of clinical management, it is understood that all administrative and clinical aspects must be aligned with the specific needs of Indigenous patients, fostering care practices that are culturally sensitive beyond technical considerations. This approach is consistent with the literature, which emphasizes the importance of well-structured administrative processes to ensure the effectiveness of care^(18)^.

The research reveals that planning health actions for Indigenous populations must take into account the historical and cultural processes of this group, from territorial management to direct care, adapting to the values and ways of life of each ethnic group. This directive aligns with theoretical foundations of transcultural care, offering services based on a deep understanding of the cultural frameworks of those being cared for^(5)^, and guided by the principles of clinical management, enabling broader and more effective approaches^(6)^. Integrating these diversities into the operational models of *“SUS”* can enhance the responsiveness of services, promoting a more inclusive, equitable, and effective approach within the hospital context^(19)^.

In this setting, case management, a micro-management technology within clinical care, may represent a promising strategy for addressing the challenges revealed in the care of Indigenous individuals. The case manager acts as an advocate for the patient and their family, facilitating communication with service providers, coordinating care across the healthcare network, and ensuring that the care plan is continuously followed and monitored^(6)^.

Understanding the nuances that influence both the demand for and the provision of healthcare to Indigenous populations is of utmost importance for building bonds of trust and partnership, foundations for more humanized and personalized care^(20)^. Within this framework, the case manager brings together the various professionals involved, reinforcing interprofessional collaboration as a strategy to confront inequalities and barriers to access^(6)^. Their role can enhance service integration, continuity of care, and the overall patient experience by adapting to the cultural and contextual specificities of Indigenous peoples, thus playing a strategic role in Indigenous healthcare delivery.

However, the lack of specific training to operate in transcultural contexts represents a significant barrier, as evidenced in this study by reports of difficulties in cultural integration and professional adaptation to Indigenous care. This gap in cultural competence undermines communication and contributes to the distancing between health services, patients, and families^(21)^, highlighting the need for a care model that values and integrates the cultural practices of Indigenous patients.

In light of clinical management, cultural adaptability emerges as essential to the provision of care, requiring the inclusion of training focused on interculturality and the reformulation of clinical processes to incorporate Indigenous practices and traditions, overcoming resistance and prejudice. In this regard, strengthening professional training policies, with an emphasis on intercultural competencies and interprofessional collaboration, is fundamental to consolidating a more inclusive and effective clinical management approach.

Health professionals must therefore develop the skills and competencies needed to assess and respect the cultural, ethnic, and social aspects of individuals in their particular contexts, enabling communication that supports accurate diagnosis and treatment, conflict management, and improved treatment adherence^(22)^. Valuing Indigenous culture contributes to a more harmonious relationship between professionals and patients, enhancing the quality of care provided. Beyond the structural and communicational challenges observed, it is important to recognize that cultural values related to care vary among different Indigenous ethnic groups. Given this diversity, it is essential that professionals understand the ethnic-cultural frameworks that guide care practices prior to entering Indigenous territories. These variations include, for example, contexts in which gender roles dictate that communication with health professionals be conducted preferably by men, as well as differences in the meanings attributed to food, hygiene, and comfort, all of which must be respected in the organization of care^(23)^.

However, the lack of awareness or the devaluation of these practices, as evidenced in this study, can weaken the health services provided and lead to potential complications in treatment adherence during hospitalization^(18,24)^. Therefore, it is necessary to adopt strategies that integrate cultural knowledge into clinical practices, grounded in interprofessional dialogue and the joint planning of actions aligned with the principles of clinical management, the regionalization of the *“SUS”*, and the protection of Indigenous peoples’ rights.

Within this context, hospital care for Indigenous populations faces specific challenges, such as cultural and linguistic barriers that hinder communication between Indigenous patients, their families, and healthcare professionals. These obstacles may compromise the quality of care and further exacerbate the situation, especially considering the structural issues faced by many hospitals, including inadequate services, lack of resources and proper equipment, high staff turnover, and excessive workloads^(6)^. Additionally, prolonged hospital stays and frequent admissions due to acute or chronic conditions are factors that intensify the suffering of Indigenous patients, who often feel isolated from family life and concerned about the relatives who remain in their communities. This emotional distress can negatively impact the recovery process^(16,25)^.

In this context, clinical management plays a central role in Indigenous healthcare, being essential to ensure quality care that respects the cultural diversity of patients, promotes community participation, and guarantees equitable access according to the actual needs of these populations. Guiding care practices by such principles helps to avoid stereotypes that label Indigenous individuals as “difficult to deal with”, recognizing that different cultures may assign distinct meanings to similar situations^(16)^.

However, health management often views these issues as problematic, mainly due to the persistence of the biomedical model, which hinders the flexibility required for intercultural dialogue in this type of care^(18)^. The traditional approach, predominantly focused on technical and clinical aspects, neglects the cultural complexity and specific needs of Indigenous patients, resulting in care that disregards their cultural and social particularities, thereby limiting the effectiveness of treatment and adherence. In this regard, guidance based on the principles of clinical management, by emphasizing comprehensive care, coordination across different levels of attention, and the personalization of health practices^(6)^, can offer a broader and more adaptive approach. This contributes to improving care practices in hospital settings and promotes care that is more sensitive and appropriate to the cultural needs of Indigenous peoples.

From the perspective of clinical management, alignment with Indigenous health policies, such as the *“PNASPI”*, facilitates the implementation of specialized care that respects and integrates the cultural practices of Indigenous peoples. Health professionals, by recognizing the importance of differentiated care and the ability to work in transcultural contexts, can offer more equitable and inclusive healthcare^(24)^. This approach can drive improvements in communication and treatment adherence, strengthening the trust between patients, their communities, and health services, and contributing to the promotion of more equitable care experiences centered on the actual needs of Indigenous individuals.

There is evidence that exposure to interculturality during professional training better prepares healthcare workers to address the cultural specificities of Indigenous patients^(24)^. Developing the ability to work in transcultural contexts is essential for professionals involved in Indigenous care^(24)^. These studies align with the experiences reported by the professionals in this research, reinforcing the importance of engaging with interculturality from the outset of training, preparing professionals to face the cultural and communicational challenges that arise in Indigenous healthcare. Therefore, the discussion of the findings through the lens of clinical management highlights the importance of a coordinated, interprofessional care model, sensitive to diversity and aligned with the regionalization guidelines of the *“SUS”*. Building *“RAS”* (Health Care Networks) that engage with Indigenous territories, combined with the training of professionals equipped to work in intercultural contexts, has the potential to transform the hospital care experience of Indigenous peoples in Brazil.

### Study limitations

The main limitation of this study is the lack of proper systematization of records related to Indigenous patient care, which compromises the depth of the investigation into hospital-based assistance. The absence of complete and well-documented data limits the ability to conduct a detailed analysis of practices and the challenges encountered. Furthermore, the study was conducted in a specific university hospital, which may restrict the generalizability of the findings to other institutions or contexts, given the cultural diversity and variations in health practices among different Indigenous populations. These factors may influence the results and limit the applicability of the conclusions in broader settings.

Acknowledging these limitations, the study focused on carefully exploring relevant issues and deepening the understanding of professionals’ experiences with a history of providing care to Indigenous patients. This approach enabled a richer and more detailed comprehension of the practices and challenges faced, despite the constraints imposed by the lack of systematized data and the specific context of the research.

### Contribution to the field of study

The study offers important contributions to the field of Nursing by highlighting the complexity of caring for Indigenous individuals in hospital settings, underscoring the need for a more sensitive approach adapted to the cultural specificities of these patients. In this context, it emphasizes the importance of incorporating theories into educational curricula that support culturally sensitive and humanized care practices, contributing to the preparation of professionals capable of working in diverse sociocultural environments.

Furthermore, the study underscores the relevance of clinical management, supported by theoretical frameworks that recognize care as a relational and culturally situated phenomenon, as well as the use of case management as a strategic micro-management tool in the care of Indigenous populations. These approaches reinforce the importance of coordination and management competencies among nurses, which are essential to ensuring continuity, comprehensiveness, and quality of care. Finally, the study advocates for the adaptation of health policies to incorporate cultural diversity, arguing that intercultural experiences during professional training, combined with evidence-based practices, can enhance the quality of care provided.

## FINAL CONSIDERATIONS

Based on the experiences of hospital-based healthcare professionals, this study revealed significant challenges in the care of Indigenous populations, particularly regarding care coordination and continuity, as well as the cultural adaptation of services provided. Linguistic barriers, the lack of adequate intercultural training, and weaknesses in the articulation between levels of care and Indigenous territories were identified as obstacles to delivering comprehensive and culturally sensitive care. In this context, clinical management emerges as a strategic possibility to be explored, especially when associated with approaches that recognize care as a relational and culturally situated practice. The adoption of strategies such as case management and network-based coordination, grounded in frameworks that value the understanding of culturally embedded meanings of care, can enhance the quality of healthcare provided to Indigenous populations, promoting greater equity and continuity.

The findings of this study reinforce the need to strengthen the intercultural training and preparation of healthcare professionals, as well as to promote strategies that support care transitions in a coordinated and respectful manner, tailored to the specificities of each ethnic group. In this way, the study points to pathways that should be further explored by future research, aiming to assess the applicability of culturally congruent and humanized care models aligned with the values, expectations, and contexts of Indigenous peoples.

## VAILABILITY OF DATA AND MATERIAL

The research data are available within the article.
