# Supplementary material for: Experiences in the care of Indigenous people in hospital settings: challenges for clinical management
Source: Rev Bras Enferm. 2026 Mar 16;79:e20240467. doi: 10.1590/0034-7167-2024-0467 (PMC12991436; doi:10.1590/0034-7167-2024-0467)
Supplement: Translation

# Translation: Experiências no cuidado à pessoa indígena na atenção hospitalar: desafios à gestão da clínica

**DOI:** 10.1590/0034-7167-2024-0467pt

**Authors:** Alidio Vieira Nunes Duarte, Vanessa Alves Mendes, Carolina Giordani da Silva, Mara Regina Rosa Ribeiro, Gímerson Erick Ferreira

**Affiliations:** I Hospital Universitário Júlio Müller. Cuiabá, Mato Grosso, Brasil; II Universidade Federal de Mato Grosso. Cuiabá, Mato Grosso, Brasil

**Keywords:** Gestão Clínica, Saúde de Populações Indígenas, Integralidade em Saúde, Cultura Indígena, Pessoas Indígenas.

## Abstract

**Objetivos:**

analisar a experiência de profissionais de saúde da atenção hospitalar no cuidado à pessoa indígena, sob a perspectiva da gestão da clínica.

**Métodos:**

estudo qualitativo, descritivo-exploratório, com entrevistas orientadas pela Técnica do Incidente Crítico, com 18 profissionais de um hospital universitário federal da região Centro-Oeste do Brasil. Os dados foram analisados com auxílio do *software* IRaMuTeQ^®^ e análise de conteúdo temática.

**Resultados:**

emergiram cinco classes semânticas organizadas em duas categorias, evidenciando desafios como barreiras culturais, linguísticas e falta de formação intercultural que comprometem a continuidade do cuidado e a comunicação com o paciente, fragilizando a implementação da gestão da clínica.

**Considerações Finais:**

a gestão da clínica, aliada às especificidades culturais, mostra-se estratégica para qualificar o cuidado hospitalar à população indígena. O estudo contribui à Enfermagem, destacando a importância da formação intercultural e da coordenação do cuidado, favorecendo abordagens integradas, equitativas, responsivas e culturalmente sensíveis às reais necessidades desses povos.

## INTRODUÇÃO

A população indígena no Brasil corresponde a aproximadamente 0,83% da população total, com cerca de 1.693.535 pessoas^(1)^. Essa população vivencia situações de desigualdade social diversas, tendo o acesso universal e equânime aos serviços de assistência à saúde ainda como um desafio^(2)^.

Apesar dos avanços nos últimos anos com a implantação da Política Nacional de Atenção à Saúde dos Povos Indígenas (PNASPI), permanecem os entraves nas pautas das discussões sobre as reais necessidades de saúde desta população. Problemas como o acolhimento inadequado e a falta de continuidade do vínculo com a rede de atenção à saúde ainda são evidentes^(3)^. Esta conjuntura destaca a necessidade urgente de um debate aprofundado acerca da gestão da saúde da população indígena no Sistema Único de Saúde (SUS), especificamente no que tange ao acesso a serviços de média e alta complexidade.

Em se tratando de atenção hospitalar, é imperativo adequar os serviços à realidade da população indígena, reconhecendo aspectos legítimos e singulares às suas necessidades e determinantes de saúde, com integração dos sistemas em redes e articulação com os territórios onde vivem. Nesse contexto, percebem-se movimentos propositivos na atenção à saúde indígena que coadunam com princípios para a gestão da clínica^(4)^, a exemplo do apoio matricial especializado e do projeto terapêutico singular, tecnologias com potencial estratégico para a prática da equipe de referência. Tais movimentos dialogam com a Teoria do Cuidado Transcultural, de Madeleine Leininger, que propõe a construção de ações de cuidado fundamentadas nos referenciais culturais do ser cuidado, favorecendo práticas congruentes às múltiplas demandas de saúde^(5)^.

Associadas, essas abordagens sustentam uma concepção ampliada do cuidado hospitalar indígena, que ultrapassa a dimensão biológica e incorpora elementos subjetivos, culturais e sociais, promovendo a continuidade e a integralidade da atenção nos diferentes pontos da Rede de Atenção à Saúde (RAS)^(6)^. Tal perspectiva exige profissionais capacitados para atuar em contextos culturalmente diversos, o que se mostra essencial à qualidade da assistência^(7)^.

Nesse sentido, tem-se na gestão da clínica, modelo de atenção idealizado no Brasil por Eugênio Vilaça Mendes, oportunidade ímpar de dirimir as lacunas que permeiam a atenção hospitalar à pessoa indígena. Isso porque o modelo propõe um sistema de tecnologias de microgestão aplicáveis ao SUS^(6)^, o qual fundamenta-se na articulação entre gestão, assistência e educação para a consolidação de suas práticas, e que norteia-se por princípios que consideram orientação para as necessidades de saúde e integralidade do cuidado, garantia de qualidade e segurança no atendimento, valorização de saberes e práticas diversas para resolver problemas de saúde, compartilhamento de poder e corresponsabilização entre os envolvidos na produção da saúde, educação contínua para indivíduos e organizações, foco em resultados que melhorem a saúde a qualidade de vida, e compromisso com a transparência e responsabilidade coletiva^(8)^.

Nos casos mais complexos de atenção à saúde de pessoas indígenas, os serviços são frequentemente referenciados para outros pontos da RAS, localizados em centros urbanos, que atuam como complementares à atenção oferecida nas aldeias^(9)^. Contudo, diversas condições de vulnerabilidade vivenciadas pelos povos indígenas limitam a efetividade da atenção à saúde, seja por questões organizacionais, geográficas e/ou culturais, como dificuldades de transporte, barreiras linguísticas, falta de infraestrutura adequada, escassez de profissionais capacitados, dentre outros^(10)^.

Logo, para que os serviços de saúde integrem práticas tradicionais e saberes indígenas, e para que os diversos serviços da RAS possam apreender e incluir em suas práticas as reais necessidades desta população, é fundamental a articulação entre os saberes indígenas e biomédicos^(11)^, essenciais à garantia da integralidade, equidade e à efetividade da atenção. Diante do exposto, emerge o questionamento central deste estudo: Como as experiências dos profissionais da atenção hospitalar no cuidado à pessoa indígena podem ser analisadas à luz da gestão da clínica?

## OBJETIVOS

Analisar a experiência de profissionais de saúde da atenção hospitalar no cuidado à pessoa indígena na perspectiva da gestão da clínica.

## MÉTODOS

### Aspectos éticos

O presente estudo integra o projeto matricial intitulado “Artefatos para implementação da gestão da clínica em hospital universitário”, aprovado no Comitê de Ética em Pesquisa (CEP) local, sob parecer: 6.080.13 e registro CAAE 09495919900005541 na Plataforma Brasil. O estudo segue as diretrizes da Resolução nº 466, de 12 de dezembro de 2012, do Conselho Nacional de Saúde. A participação foi voluntária e ocorreu mediante assinatura do Termo de Consentimento Livre e Esclarecido (TCLE). Para assegurar o sigilo e anonimato dos participantes, suas falas foram codificadas com a letra E seguida por um número cardinal.

### Tipo de estudo

Trata-se de um estudo descritivo-exploratório, com abordagem qualitativa por visar a compreensão do fenômeno humano através dos significados, motivos, aspirações, crenças, valores e atitudes^(12)^. O artigo segue as diretrizes do guia *Consolidated Criteria for Reporting Qualitative Research (COREQ)*.

### Cenário do estudo

O estudo foi conduzido em um Hospital Universitário Federal (HUF), gerido pela Empresa Brasileira de Serviços Hospitalares (EBSERH), localizado na região Centro-Oeste do Brasil. As unidades selecionadas para a pesquisa foram a clínica de internação materno-infantil e os ambulatórios (pediatria, doenças infectocontagiosas, feridas e estomaterapia), em virtude de estas possuírem maior demanda de atendimento a pessoas indígenas.

### Fonte de dados

Participaram do estudo 18 profissionais de saúde da equipe multiprofissional, sendo estes enfermeiros, médicos, técnicos de enfermagem, psicólogo, nutricionista e assistente social, todos com experiência no atendimento à pessoa indígena. A amostra foi selecionada por meio da técnica de amostragem *snowball* (bola de neve), na qual participantes-referência indicam outros participantes^(13)^. Os critérios de inclusão foram: profissionais da equipe multiprofissional com vínculo funcional ativo e experiência no atendimento a pessoas indígenas nas clínicas e ambulatórios do referido hospital. Foram excluídos os profissionais em férias ou afastados por qualquer motivo durante o período de coleta de dados.

### Coleta e organização dos dados

Os dados foram coletados entre julho e agosto de 2023, por meio de entrevistas semi-estruturadas, norteadas pela Técnica do Incidente Crítico (TIC). Esta técnica é amplamente utilizada em estudos da área da saúde por possibilitar que o entrevistado recupere memórias de fatos e observações importantes, facilitando a compreensão de comportamentos e vivências^(14)^.

As entrevistas foram registradas em áudio e conduzidas por um dos pesquisadores, especialista em saúde indígena, com duração média de 15 minutos. O material empírico foi transcrito integralmente utilizando a ferramenta de transcrição *online* do *Microsoft Word^®^* e posteriormente processado no *software* IRaMuTeQ^®^ (*Interface de R pour les Analyses Multidimensionnelles de Textes et de Questionnaires*), utilizado como apoio à análise qualitativa. Para a construção das classes, foram adotados o Método de Reinert, com aproveitamento de 80% do *corpus* textual, resultando no agrupamento de 196 segmentos textuais extraídos das transcrições, e o Método de Classificação Hierárquica Descendente (CHD), que gerou classes semanticamente relacionadas, representadas hierarquicamente no dendrograma (Figura 1). Essa estrutura orientou a sistematização dos conteúdos, tendo o foco analítico recaído sobre a interpretação dos sentidos presentes nos discursos, o que subsidiou a construção das categorias temáticas centrais.

**Figura 1:** Classes semânticas relacionadas às experiências no cuidado à pessoa indígena, 2023

### Análise dos dados

A análise dos dados foi realizada por meio da Análise de Conteúdo Temática^(15)^, seguindo três etapas: I) organização dos dados, II) exploração do material, com agrupamento das informações em categorias, e III) reflexão crítica para a interpretação dos dados à luz do referencial teórico. Esta abordagem permitiu a identificação das experiências dos profissionais de saúde no atendimento à pessoas indígenas no ambiente hospitalar sob a perspectiva da gestão da clínica.

## RESULTADOS

Entre os participantes do estudo, 78% (14) eram do sexo feminino e 22% (4) masculino, com faixa etária entre 31 a 40 anos (44,44%). Em relação ao perfil profissional, 22,22% (4) eram médicos, 33,33% (6) enfermeiros, 27,78% (5) técnicos de enfermagem, 5,56% (1) psicólogo, 5,56% (1) assistente social e 5,56% (1) nutricionista. O nível de escolaridade predominante foi o de nível superior com especialização *Lato Sensu* (50%). O tempo de formação profissional variou, com 38,89% possuindo entre 11 e 15 anos de experiência. Os participantes atuavam principalmente em unidades de ambulatórios (38,89%) e na clínica pediátrica (22,22%).

A análise qualitativa dos dados, apoiada pelo *software* IRaMuTeQ, gerou cinco classes organizadas em dois eixos, cujas narrativas dos profissionais permitiram a construção de duas categorias temáticas centrais, relacionados aos desafios presentes em suas experiências no cuidado à pessoa indígena: “Coordenação e continuidade do cuidado”, e “Integração cultural e adaptação dos processos assistenciais”. A Figura 1 ilustra as relações entre essas classes e os eixos temáticos identificados. Destaca-se que, embora o processamento no *software* tenha auxiliado na organização dos dados, a análise focou nos sentidos expressos pelos participantes, não se limitando à frequência ou à distribuição dos segmentos textuais.

Assim, desta análise emergiram duas categorias, sendo a primeira intitulada “Desafios na coordenação e continuidade do cuidado à pessoa indígena”. Nesta categoria, os discursos dos profissionais revelam os principais obstáculos enfrentados para garantir a continuidade do cuidado à população indígena, sobretudo nos aspectos relacionados à comunicação intercultural e à articulação entre os diferentes níveis de atenção da RAS. Os relatos indicam a presença de barreiras linguísticas e culturais, somadas à fragilidade na troca de informações e à ausência de fluxos coordenados de cuidados, especialmente na transição entre os serviços de atenção primária e terciária. Chamam a atenção narrativas nas quais o homem indígena é o principal elo de comunicação, predomínio hierárquico comum em algumas etnias devido ao seu domínio de outras línguas, o que limita ainda mais a comunicação com o paciente, comprometendo a compreensão de suas reais necessidades e, consequentemente, a qualidade do cuidado e equidade na assistência. No Quadro 1, apresentam-se os segmentos de texto representativos que ilustram as subcategorias emergentes.

**Quadro 1:** Segmentos de texto representativos da Categoria 1 “Desafios na coordenação e continuidade do cuidado à pessoa indígena”, por classe semântica

CATEGORIA 1 - “Desafios na coordenação e continuidade do cuidado à pessoa indígena”	
Subcategoria I (classes 2 e 3): Necessidades de adaptar as práticas de comunicação. Dificuldades na transição do cuidado e na continuidade da assistência entre diferentes pontos da rede de atenção.	*O paciente, com seu acompanhante, os dois eram indígenas e só se comunicavam entre si e não entendia o que a gente falava e a gente também não entendia o que eles falavam. Então isso gerou uma dificuldade no nosso trabalho.* (E04)
	[...] *quando tinha dificuldade na comunicação e acho que isso prejudicava bastante o atendimento.* (E03)
	[...] *eu acho que facilitaria muito o nosso trabalho e a vida deles se eles tivessem um apoio local, mais organizado, entendeu? Se tivesse uma forma de se comunicar com o médico, com enfermeiro lá do local, se a gente pudesse ter essa comunicação entre o profissional daqui da rede terciária com a rede básica* [...]. *Facilitaria muito o atendimento dessas pessoas, porque eu poderia passar a recomendação para esse profissional da rede básica, e ele faria a assistência direta ao paciente, lá na região deles.* (E11)
	*A dificuldade que a gente tem com os indígenas, porque quando eles internam no hospital, normalmente não conseguem entender o português, tem uma certa dificuldade em entender os cuidados.* (E13)
Subcategoria II (classe 5): Importância dos aspectos procedimentais e administrativos na implementação e coordenação do cuidado.	*A mãe, ela não falava com a gente, e o pai era muito difícil, ele não aceitava o tratamento e com muito custo, conversando com ele* […]. (E10)
	*O pai veio junto, mas no momento que a gente precisou fazer procedimento na criança urgente a enfermeira não conseguia porque a mãe não permitia, somente com autorização do pai.* (E17)
	*A mãe não permitia porque o pai não estava presente. Ela não podia autorizar sem a presença do pai.* (E17)

A segunda categoria, intitulada “Desafios na integração cultural e adaptação aos processos de cuidado à pessoa indígena”, abrange os sentidos atribuídos pelos profissionais às dificuldades em incorporar elementos da cultura indígena na assistência hospitalar. As narrativas evidenciam a escassez de formação específica para o cuidado a essa população, bem como sentimentos de insegurança, descontentamento e até impotência diante da falta de preparo institucional para lidar com as diferenças culturais. Em muitos casos, a assistência prestada segue modelos padronizados, desconsiderando os valores, crenças e práticas tradicionais dos pacientes indígenas. Os trechos destacados no Quadro 2 revelam a complexidade dessas vivências e como elas impactam tanto a qualidade do cuidado quanto a relação entre profissional e paciente.

**Quadro 2:** Segmentos de texto representativos da Categoria 2 “Desafios na integração cultural e adaptação aos processos de cuidado à pessoa indígena”, por classe semântica

CATEGORIA 2 - “Desafios na integração cultural e adaptação aos processos de cuidado à pessoa indígena”	
Subcategoria I (classe 1): Importância de reconhecer e respeitar a cultura indígena no contexto hospitalar	*Então eles têm uma cultura diferente da nossa e às vezes a gente tem dificuldade em adaptar a rotina do hospital, a essa cultura deles.* (E05)
	*Cuidamos como se fosse paciente conforme nossa rotina, mas a gente não tem aquela capacitação, aqueles cuidados específicos, para paciente indígena* [...] *é bem diferente da nossa cultura.* (E05)
	*Você percebe que ele não demonstra uma compreensão do que está sendo proposto e a equipe, em se tratando da equipe profissional, também não tem um feeling para ter uma* [compreensão]. (E09)
Subcategoria II (classe 4): Desafios dos profissionais em prestar assistência adequada	*Me marcou bastante porque não tivemos tanto apoio e não sei nem se é porque a instituição não quis dar apoio.* (E05)
	*Eu percebi, o comportamento das pessoas, não chega a ser negligência, mas um cuidado superficial, cuidado menos comunicativo, um cuidado grosso modo, mais seco, pouca conversa, pouca interação com o paciente.* (E09)
	*A gente tem muita dificuldade de entendimento na consulta, nem sempre aquilo que eu recomendo da receita, eu me sinto ainda insegura se eles realmente estão entendendo aquilo que eu estou passando.* (E11)
	*A gente não entende muito bem a cultura deles, às vezes a língua e a gente acaba se sentindo impotente.* (E10)

## DISCUSSÃO

A atenção à saúde indígena no ambiente hospitalar é marcada por desafios complexos enfrentados pelos profissionais de saúde, refletindo a necessidade de uma abordagem mais integrada e adaptada às especificidades culturais da população indígena. Estes desafios transcendem o acesso básico à saúde, abrangendo aspectos fundamentais da comunicação intercultural e da coordenação do cuidado, elementares para garantir atendimento integral e de qualidade^(16)^. Nesse contexto, a gestão da clínica demanda a atuação de equipes interprofissionais articuladas, capazes de integrar saberes distintos e compartilhar responsabilidades no cuidado à população indígena^(6)^.

Os resultados da pesquisa destacam que a comunicação eficaz e a coordenação entre diferentes níveis de cuidado são aspectos determinantes para a efetividade da atenção hospitalar à população indígena. A análise revelou dificuldades na transição do cuidado e na continuidade da assistência, evidenciadas pela complexidade retratada pelos participantes na comunicação entre os diversos serviços de saúde. À luz da gestão da clínica, estes aspectos são necessários para assegurar qualidade assistencial e continuidade do cuidado, exigindo uma atuação interprofissional integrada, capaz de promover o intercâmbio de informações e a adequação das condutas às especificidades culturais dos pacientes. Esse processo envolve o suporte de navegação pelo sistema de saúde, abrangendo desde a coordenação clínica, com encaminhamentos e transferências de informações, à coordenação logística, com apoio, transporte e questões financeiras^(6)^.

Desse modo, torna-se imprescindível a implementação de estratégias para superar barreiras linguísticas e culturais, como o uso de tradutores e a adaptação de materiais de comunicação, considerando as particularidades culturais e linguísticas de cada etnia indígena. A produção de materiais educativos bilíngues, com a participação de profissionais indígenas e lideranças comunitárias, pode representar uma medida concreta e viável no contexto hospitalar. Tais iniciativas contribuem para aprimorar a comunicação entre os diferentes níveis de atenção, favorecendo a transferência adequada de informações e responsabilidades, e assegurando que o acompanhamento da pessoa indígena ocorra de forma coordenada e culturalmente sensível.

No entanto, ao abordar a assistência à população indígena em nível hospitalar, os resultados deste estudo apontam para lacunas na coordenação do cuidado, particularmente na transição entre diferentes níveis de atenção e na contrarreferência para as aldeias ou territórios. Essa ausência de mecanismos de coordenação pode impactar negativamente a continuidade do cuidado, dificultando a integração entre os serviços hospitalares e a rede de saúde local. Tal cenário reforça a necessidade de estratégias propositivas de articulação em rede com foco na implementação de cuidados transicionais sensíveis e congruentes às especificidades culturais de cada etnia indígena^(5,17)^, e reafirma a importância do trabalho interprofissional como elemento central na gestão clínica.

Para enfrentar as lacunas identificadas, a gestão da clínica emerge como modelo potencialmente eficaz visto que seus princípios orientadores têm alicerce em diretrizes e protocolos que facilitam a comunicação e a integração entre os serviços, nos mais diversos pontos de atenção da RAS^(4)^. Em tese, o modelo promove a criação de equipes dedicadas e a inclusão das comunidades indígenas na elaboração e monitoramento das estratégias, alinhando-se à necessidade de diretrizes clínicas específicas para melhorar a coordenação e o cuidado contínuo.

Sob a perspectiva da gestão da clínica, entende-se que todos os aspectos administrativos e clínicos devem estar alinhados às necessidades específicas dos pacientes indígenas, promovendo práticas de cuidado que sejam culturalmente sensíveis, para além dos aspectos técnicos. Tal perspectiva expressa consonância com a literatura, que ressalta a importância de processos administrativos bem estruturados para assegurar a efetividade do cuidado^(18)^.

A pesquisa revela que o planejamento das ações em saúde indígena deve considerar os processos históricos e culturais deste grupo populacional, desde a gestão territorial até a assistência direta, adaptando-se aos valores e modos de vida de cada etnia. Essa diretriz está em consonância com fundamentos teóricos do cuidado transcultural, com a oferta de cuidados pautados na compreensão profunda dos referenciais culturais do ser cuidado^(5)^, e orientados por princípios da gestão da clínica, possibilitando abordagens mais amplas e resolutivas^(6)^. A integração dessas diversidades aos modelos operacionais do SUS pode ampliar a capacidade de resposta dos serviços, promovendo uma abordagem mais inclusiva, equitativa e resolutiva no contexto hospitalar^(19)^.

Nesse contexto, a gestão de caso, tecnologia de microgestão da clínica, pode representar uma estratégia promissora no equacionamento dos desafios desvelados na atenção à pessoa indígena. O gestor de caso atua como advogado do paciente e de sua família, facilitando a comunicação com prestadores de serviços, coordenando o cuidado na rede assistencial e assegurando que o plano de cuidado seja seguido e monitorado continuamente^(6)^.

Compreender as nuances que influenciam a procura e a oferta de atendimento à pessoas indígenas é de suma importância para a construção de vínculos de confiança e parceria, base para um cuidado mais humanizado e personalizado^(20)^. Nesse contexto, o gestor de caso articula os diferentes profissionais envolvidos, reforçando o trabalho interprofissional como estratégia para enfrentar desigualdades e barreiras no acesso^(6)^. Sua atuação pode favorecer a integração dos serviços, a continuidade do cuidado e a melhoria da experiência do paciente, ao adaptar-se às especificidades culturais e contextuais dos povos indígenas, desempenhando, assim, papel estratégico na assistência à população indígena.

No entanto, a falta de formação específica para atuar em contextos transculturais representa uma barreira significativa, constatada neste estudo pelos relatos de dificuldades na integração cultural e adaptação dos profissionais ao cuidado de pessoas indígenas. Essa lacuna de competência cultural compromete a comunicação e contribui para o distanciamento entre os serviços de saúde, pacientes e familiares^(21)^, o que sugere a necessidade de um modelo de atendimento que valorize e integre as práticas culturais dos pacientes indígenas.

À luz da gestão da clínica, a adaptabilidade cultural revela-se essencial à prestação de cuidados, exigindo a inclusão de capacitações voltadas à interculturalidade e à reformulação dos processos clínicos para a incorporação das práticas e tradições indígenas, superando resistências e preconceitos. Nesse sentido, o fortalecimento das políticas de formação profissional, com ênfase em competências interculturais e atuação interprofissional, é fundamental para consolidar uma gestão da clínica mais inclusiva e resolutiva.

Os profissionais de saúde devem, portanto, desenvolver habilidades e competências para avaliar e respeitar os aspectos culturais, étnicos e sociais dos indivíduos em suas particularidades, permitindo uma comunicação que favoreça o diagnóstico e tratamento adequados, o gerenciamento de conflitos e a melhoria na adesão ao tratamento^(22)^. Valorizar a cultura indígena contribui para uma relação mais harmoniosa entre profissionais e pacientes, qualificando o cuidado prestado. Para além das dificuldades estruturais e comunicacionais observadas, é importante considerar que os valores culturais relacionados ao cuidado variam entre os diferentes grupos étnicos indígenas. Dada a diversidade existente, é essencial que os profissionais compreendam previamente os referenciais étnico-culturais que orientam as práticas de cuidado antes mesmo de ingressarem nos territórios indígenas. Essas variações incluem, por exemplo, contextos em que os papeis de gênero determinam que a comunicação com profissionais de saúde seja realizada preferencialmente por homens, além de diferenças nos significados atribuídos à alimentação, higiene e conforto, que devem ser respeitadas na organização do cuidado^(23)^.

Entretanto, o desconhecimento ou a desvalorização dessas práticas, como evidenciados neste estudo, podem fragilizar os serviços de saúde prestados e culminar em possíveis complicações no processo de adesão ao tratamento durante a hospitalização^(18,24)^. Assim, é necessário adotar estratégias que integrem o conhecimento cultural às práticas clínicas, fundamentadas no diálogo interprofissional e no planejamento conjunto de ações alinhadas aos princípios da gestão da clínica, da regionalização do SUS e da garantia dos direitos dos povos indígenas.

Nesse panorama, o atendimento à população indígena na atenção hospitalar enfrenta desafios específicos, como barreiras culturais e linguísticas, que dificultam a comunicação entre pacientes indígenas, seus familiares e profissionais de saúde. Esses obstáculos podem comprometer a qualidade do cuidado e agravar ainda mais a situação, especialmente considerando os problemas estruturais enfrentados por muitos hospitais, como a precariedade dos serviços, a falta de recursos e equipamentos adequados, a alta rotatividade de profissionais e cargas excessivas de trabalho^(6)^. Adicionalmente, o tempo prolongado de permanência no hospital e as internações frequentes por condições agudas ou crônicas são fatores que intensificam o sofrimento dos pacientes indígenas, que comumente se sentem isolados da vida familiar e preocupados com os membros da família que permanecem na comunidade, o que pode impactar negativamente no processo de recuperação^(16,25)^.

Nesse contexto, a gestão da clínica desempenha papel central na assistência à saúde indígena, sendo necessária para garantir cuidados de qualidade que respeitem a diversidade cultural dos pacientes, promovam a participação comunitária e assegurem acesso equitativo, conforme as reais necessidades destas populações. A orientação das práticas de cuidado por tais diretrizes ajuda a evitar estereótipos que rotulam os indígenas como “difíceis de lidar”, reconhecendo que diferentes culturas podem atribuir significados distintos a situações semelhantes^(16)^.

No entanto, a gestão da saúde muitas vezes encara essas questões como problemáticas, principalmente devido à persistência do modelo biomédico, o que dificulta a flexibilidade necessária para o diálogo intercultural neste tipo de assistência^(18)^. A abordagem tradicional, centrada predominantemente em aspectos técnicos e clínicos, negligencia a complexidade cultural e as necessidades específicas dos pacientes indígenas, resultando em um atendimento que desconsidera as particularidades culturais e sociais dos pacientes, limitando a eficácia do cuidado e a adesão ao tratamento. Nesse sentido, a orientação por princípios da gestão da clínica, ao enfatizar a integralidade do cuidado, a articulação entre diferentes níveis de atenção e a personalização das práticas de saúde^(6)^, pode oferecer uma abordagem mais abrangente e adaptativa, contribuindo para a melhoria das práticas de cuidado na atenção hospitalar e promovendo um atendimento mais sensível e adequado às necessidades culturais das pessoas indígenas.

Sob a perspectiva da gestão da clínica, o alinhamento com políticas de saúde indígena, como a PNASPI, facilita a implementação de uma atenção especializada que respeite e integre as práticas culturais dos povos indígenas. Os profissionais de saúde, ao reconhecerem a importância da atenção diferenciada e da capacidade de trabalhar em contextos transculturais, podem oferecer um cuidado mais equitativo e inclusivo^(24)^. Essa abordagem pode ser propulsora de melhorias na comunicação e na adesão ao tratamento, fortalecendo a relação de confiança entre os pacientes e suas comunidades com os serviços de saúde, e contribuindo para a promoção de experiências de cuidado mais equitativas, centradas nas reais necessidades da pessoa indígena.

Há evidências de que a experiência com a interculturalidade desde a formação dos profissionais pode melhor preparar para o atendimento às especificidades culturais dos pacientes^(24)^, sendo o desenvolvimento da capacidade de trabalhar em um contexto transcultural indispensável para os profissionais de saúde no cuidado à pessoa indígena^(24)^. Estes estudos demonstram convergência com as experiências relatadas pelos profissionais desta pesquisa, reforçando a importância de vivenciar a interculturalidade desde a formação, preparando os profissionais para enfrentarem os desafios culturais e comunicacionais que surgem no atendimento a pessoas indígenas. Portanto, a discussão dos resultados à luz da gestão da clínica revela a importância de um modelo de cuidado coordenado, interprofissional, sensível à diversidade e alinhado às diretrizes da regionalização do SUS. A construção de RAS que dialoguem com os territórios indígenas, aliada à formação de profissionais capacitados para o trabalho em contextos interculturais, pode transformar a experiência de cuidado hospitalar vivenciada pelos povos indígenas no Brasil.

### Limitações do estudo

A principal limitação deste estudo é a ausência de sistematização adequada dos registros no atendimento à população indígena, o que compromete a profundidade da investigação sobre a assistência prestada no contexto hospitalar. A falta de dados completos e bem documentados limita a capacidade de análise detalhada das práticas e dos desafios enfrentados. Além disso, o estudo foi realizado em um hospital universitário específico, o que pode restringir a generalização dos resultados para outras instituições ou contextos, visto que a diversidade cultural e as variações nas práticas de saúde entre diferentes populações indígenas podem influenciar os resultados, limitando a aplicabilidade das conclusões em contextos diversos.

Reconhecendo essas limitações, o estudo se concentrou em explorar cuidadosamente as questões relevantes e em aprofundar as experiências de profissionais com histórico de atendimento a pacientes indígenas. Essa abordagem permitiu obter uma compreensão mais rica e detalhada das práticas e dos desafios enfrentados, apesar das limitações impostas pela falta de dados sistematizados e pelo contexto específico da pesquisa.

### Contribuições para a Área

O estudo oferece importantes contribuições para a Enfermagem ao destacar a complexidade do cuidado à pessoa indígena na atenção hospitalar, sublinhando a necessidade de uma abordagem mais sensível e adaptada às especificidades culturais desses pacientes. Neste contexto, destaca-se a importância de que os currículos de formação contemplem teorias que fundamentam práticas de cuidado culturalmente sensíveis e humanizadas, contribuindo para a preparação de profissionais aptos a atuarem em contextos socioculturais diversos.

Além disso, o estudo ressalta a relevância da gestão da clínica, aliada a referenciais teóricos que reconhecem o cuidado como um fenômeno relacional e culturalmente situado, bem como o uso da gestão de caso como tecnologia de microgestão estratégica no atendimento às populações indígenas. Tais abordagens reforçam a importância de competências em coordenação e gestão por parte dos enfermeiros, essenciais para assegurar a continuidade, a integralidade e a qualidade da assistência. Por fim, o estudo sugere a adaptação das políticas de saúde para incorporarem a diversidade cultural, defendendo que a vivência intercultural desde a formação, aliada a práticas baseadas em evidências, pode qualificar o cuidado prestado.

## CONSIDERAÇÕES FINAIS

A pesquisa revelou, a partir da experiência de profissionais de saúde da atenção hospitalar, desafios importantes no cuidado à população indígena, especialmente no que tange à coordenação e continuidade do cuidado, bem como à adaptação cultural dos serviços prestados. As barreiras linguísticas, a ausência de formação intercultural adequada e as fragilidades na articulação entre os níveis de atenção e os territórios indígenas foram destacadas como obstáculos à efetivação de um cuidado integral e culturalmente sensível. Nesse contexto, a gestão da clínica emerge como uma possibilidade estratégica a ser explorada, sobretudo quando associada à abordagens que reconhecem o cuidado como prática relacional e culturalmente situada. A adoção de estratégias como a gestão de caso e a articulação em rede, ancoradas em referenciais que valorizam a compreensão dos significados atribuídos ao cuidado nas diferentes culturas, pode qualificar a atenção prestada às populações indígenas, promovendo maior equidade e continuidade.

Os resultados deste estudo reforçam a necessidade de fortalecer a formação e o preparo intercultural dos profissionais, além de fomentar estratégias que promovam a transição do cuidado de forma articulada e respeitosa às especificidades de cada etnia. Desse modo, aponta para caminhos que devem ser aprofundados por futuras pesquisas, com vistas a avaliar a aplicabilidade de modelos assistenciais culturalmente congruentes e humanizados, alinhados aos valores, expectativas e contextos dos povos indígenas.

## DISPONIBILIDADE DE DADOS E MATERIAL

Os dados de pesquisa estão disponíveis no corpo do artigo.
